# Reproducibility and relative validity of a newly developed web-based food-frequency questionnaire for assessment of preconception diet

**DOI:** 10.1186/s40795-019-0310-y

**Published:** 2019-11-07

**Authors:** L. Salvesen, E. R. Hillesund, F. N. Vik, A. L. Brantsæter, N. C. Øverby

**Affiliations:** 1 Department of Public Health, Sport and Nutrition, Faculty of Health and Sport Sciences, University of Agder, PO Box 422, 4604 Kristiansand, Norway; 2 Department of Environmental Exposure and Epidemiology, Norwegian Institute of Public Health, Oslo, Norway

**Keywords:** Reproducibility, Relative validity, Food-frequency questionnaire, Weighed food record, Preconception diet, Preconception phase, Young adults

## Abstract

**Background:**

The importance of diet and nutrition during preconception age is a window of opportunity to promote future parental and transgenerational health. As a sub-study to a large Norwegian study, ‘Diet today – health of tomorrow’, a food-frequency questionnaire (FFQ) was developed to assess diet during the preconception phase in young adults aged 20 – 30 years and in this paper we report the reproducibility and relative validity of this questionnaire.

**Methods:**

The FFQ was developed from an existing FFQ validated in adolescents. Participants were recruited on social media and at a university. Reproducibility was assessed by comparing the test and retest of the FFQ. Relative validity was assessed by comparing intake measured by the FFQ with a 7-day weighed food record. Energy, nutrients and food intake were used to assess the reproducibility and relative validity of the FFQ. The study applied the Spearman’s rank correlation coefficient, percentage of agreement and Cohen’s Kappa to assess reproducibility and validity.

**Results:**

There were 32 participants recruited to the study, of which 21 participants completed both the test-retest reproducibility and the relative validation. The test-retest reproducibility had a median correlation coefficient of 0.85 for energy and nutrients, a median Spearman’s rank correlation coefficient of 0.75 and a median Cohen’s Kappa of 0.51 for food groups. The relative validity of the FFQ had a median correlation coefficient of 0.59 for energy and nutrients, a median Spearman’s rank correlation coefficient of 0.54 and a median Cohen’s Kappa of 0.28 for food groups.

**Conclusion:**

This newly developed FFQ for preconception diet in young adults had a satisfactory test-retest reproducibility and fair relative validity.

## Background

Eating a healthy and balanced diet throughout the life course protects against malnutrition in all its forms, as well as a range of non-communicable diseases (NCDs) and conditions [1]. The understanding that diet and nutrition during the preconception phase of life is important for a future child’s development and later life conditions is a developing field of study, showing promise in promoting future parental and transgenerational health [2–6]. The preconception phase of reproductive life is defined as the time from reproductive maturity to conception [2].

The International Federation of Gynecology and Obstetrics [3] calls for worldwide action to improve diet and nutrition prior to conception in order to promote lifelong health and wellbeing, and to prevent the transmission of metabolic susceptibility to the next generation. The Lancet’s Maternal Obesity 3 [7] points out the need for future research on detailed information about specific maternal lifestyle, nutritional, and metabolic exposures that underpin effects of maternal obesity on outcomes in offspring. Assessing diet and nutritional status is therefore essential to understand how to improve the health of individuals and populations [8]. Today there is no suitable Norwegian questionnaire to approach the preconception target population, from adolescence to young adulthood. The development of a food-frequency questionnaire (FFQ) that targets this population is therefore important.

There are several dietary assessment methods, each with its strengths and limitations. The retrospective methods, such as 24-h recall, diet history and FFQs, offer a retrospective view on dietary habits and food intake. These methods rely on the participants’ memory and their ability to recall foods eaten and the frequency of intake [9]. Prospective methods assess food intake in real time, such as food records or the duplicate diet approach. These methods assess actual intake over a specific period, but are less suitable for large scale epidemiological studies, as they are time consuming, demand a high level of motivation and represent a large burden for the participants [9]. The weighed food record (WFR) should be among the first methods of choice when assessing the validity of an FFQ [10]. The prospective nature of the method reduces errors related to participants recalling food intake. Using a weighing scale to quantify the amounts of food eaten ensures accurate intake. The limitations associated with the method, such as expenses, burden for participants and social desirability bias, makes it less feasible for larger scale implementation [9, 10]. An FFQ generally consists of a fixed food list and a frequency response section and may include further details on quantity and composition. FFQs are common in large scale observational studies because they are easily administered, the least expensive and have the lowest participant burden compared with other dietary assessment methods, while being able to capture usual long-term dietary intake [11].

Given the importance of gaining more knowledge on the impact of diet and nutrition on health outcomes, it is crucial to examine the degree to which a dietary assessment method measures true intake [10, 12], by testing the validity and reproducibility. The validity is tested by comparing your method with a more reliable reference method. In order to collect data on the large population that make up preconception young adults, developing an FFQ was considered an appropriate methodological approach.

The present study is part of a larger study, ‘Diet today - health of tomorrow’. The main study aims to develop, implement and evaluate a theory and evidence based digital intervention that promotes a healthful diet preconception, optimizes fetal conditions during pregnancy, and prevents NCDs in future children. No relevant FFQ were found for the target age group in Norway, making it necessary to develop an FFQ and test its accuracy.

Therefore, the purpose of the present study was to develop an FFQ for preconception young adults and investigate its reproducibility and validity in the target population.

## Methods

### Study design and recruitment

Reproducibility was assessed by comparing a test and retest of the FFQ. Relative validity was assessed by comparing intake measured by the FFQ (test) with 7-day weighed food records. Recruitment of participants took place from November 1st until November 24th in 2017. Data were collected in the time period from November 2017 until January 2018. To be included in the study, participants had to be 20-30 years old, without children and give their consent to participate. The lower range of the age group was based on targeting young adults as they move away from home and start their independent life, thereby deciding their own diet. The upper range of the age group was based on the Norwegian age of first child birth, which were 29 for women and 32 for men in 2018, respectively [13]. The study included both preconception and periconception young adults but did not distinguish between the two. The participants needed to have access to internet, possess the necessary skills to complete an internet-based questionnaire and be willing to weigh and record their intake of food for seven consecutive days. Finally, they needed to be able to meet in person at the University of Agder, Kristiansand, Norway, at least once to attend an instructional meeting.

The study was advertised on social media, among students at the Faculty of Health and Sport Sciences at University of Agder, and through word of mouth. The advertisement led potential participants to the study website, which contained a general outline of the study, an invitation to participate, contact information, and a button for enrolment in the study. When signing up for the study, the participants gave their consent to participate and selected one of 11 possible instructional meetings to attend. As participants signed up, an e-mail containing a link to the online FFQ was sent to their e-mail address. The e-mail also contained information about the FFQ, a deadline for completing the FFQ, and information on where to meet for the instructional meeting. If a participant had not completed the FFQ 1 week before their scheduled meeting, a reminder was sent by e-mail.

For the test-retest reproducibility investigation, a link to the FFQ retest version was sent to participants via e-mail on December 13th, 2017, resulting in at least 19 days between test and retest of the FFQ.

### Study population

During the recruitment period of approximately 4 weeks, 32 participants signed up. Of these, 29 completed the first FFQ (of which three did not complete the entire FFQ). In the course of 11 instructional meetings 25 participants attended and started their 7-day WFR. Of these 25 participants, 22 completed the WFR (one participant recorded 6 days) and returned their recording booklet.

**Fig. 1:**
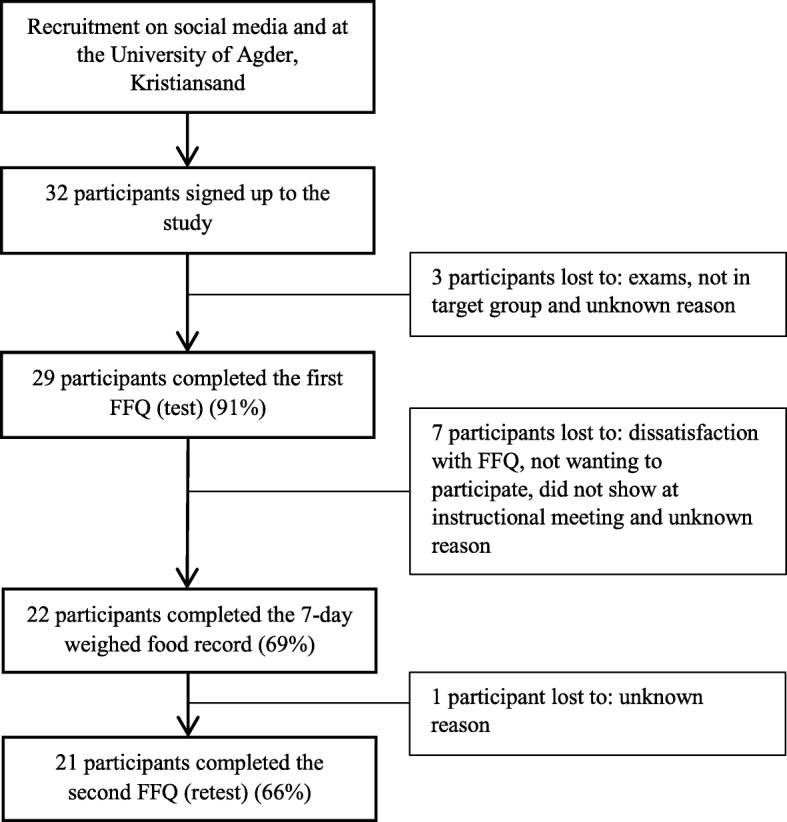
Outline of the study design and numbers participating in each study component. Number of participants (participation rate) in each study component and participants lost. Food frequency questionnaire (FFQ)

The FFQ retest was completed by 21 of the 22 participants that completed the WFR. A total of 21 participants completed all the components of the study (Fig. 1).

### The food-frequency questionnaire

The FFQ was developed from an existing FFQ targeting adolescents [14]. In the first stage of development, the original FFQ was sent to five volunteers using a purposive sampling, at which point they completed the FFQ and then answered questions according to an interview guide. Feedback from the volunteers were considered by the authors. The inputs deemed relevant based on our understanding of the age group and development of an FFQ were included in the revision of the questionnaire. In addition, the original questionnaire was revised in accordance to input from the authors and expert colleagues at the faculty. In the second stage of development, the revised version of the FFQ was sent to two new volunteers, using purposive sampling. The participants were interviewed in accordance with the interview guide upon completion of the FFQ. Based on their feedback the final version of the FFQ was created, using the online survey tool SurveyXact [15]. The food-frequency questionnaire can be found in Additional file 1. The majority of the food items in the original FFQ were included in the revised version. Some food items were excluded (food items not found relevant by the participants during the development, food items targeting children, i.e. specific sweetened breakfast cereals), and some food items were added (i.e. low- and full fat options, a wider range of condiments). Changes to frequency of intake were based on some participants requesting a greater degree of variation in order to reflect their dietary intake (i.e. adding ‘more than 3 cups per day’ for coffee).

The FFQ consisted of 146 questions aiming to reflect diet in a four-week retrospect by asking respondent to report their average intake of the specified food items during the last 4 weeks. Completing the FFQ took about 25 min. The FFQ started with the respondents’ sex, age, self-reported height and weight, and level of education, followed by 121 questions related to average consumption of foods and beverages. These were divided into different sections (beverages and dairy products, bread and grain products, lunch meats, dinner meals and side dishes, fruits and vegetables, desserts, cakes and snacks). The FFQ ended with 13 questions regarding food habits and 7 questions related to physical activity, screen time, sleep and tobacco use.

The response alternatives regarding frequency of intake varied according to food and beverage questions. For beverages and dairy products (not including yoghurt), the interval range was ‘never’, ‘1-3 per month’, ‘1-3 per week’, ‘4-6 per week’, ‘1 per day’, ‘2-3 per day’ and ‘more than 3 per day’. Dinner meals and side dishes used the interval ranges ‘never’, ‘1-3 per month’, ‘1 per week’, ‘2-4 per week’ and ‘more than 4 times per week’. Fruit and vegetables, desserts, cakes and snacks all used the interval range ‘never’, ‘1-3 per month’, ‘1 per week’, ‘2-3 per week’, ‘4-6 per week’ and ‘1 or more times per day’. The respondents reported their food intake in ‘units per month’, ‘units per week’ or ‘units per day’. The unit measurements differed between sections and foods, whereas most questions were related to a standard portion size (e.g. cup of coffee, a piece of bread, an apple). For some questions, extra information was provided (e.g. maize = 2 tablespoons or soda = 0.5 l).

Calculating food and nutrient intake was overseen by the corresponding author. All food and beverage related questions were linked to a corresponding food-code in the Norwegian food composition table [16]. The Norwegian Food Safety Authority’s “Weights, measures and portion sizes for food” [17] and the web-page “Food-Recipes” [18] were used when assigning portion sizes. Amount in grams/millilitres was calculated using portion sizes and reported frequency of intake per day (based on 1 month being 28 days, as the FFQ reported intake in a four-week retrospect). FoodCalc [19] was used to process the FFQ, based on nutritional values from the Norwegian food composition table [16]. Food and beverage items assessed by the FFQ were organized into 28 non-overlapping food groups according to nutrient profile, biological classification or culinary usage.

### The 7-day weighed food record

At the instructional meetings, the participants received general information on how and why the study was conducted, and instructions on how to weigh and record their food. They also received the equipment necessary to implement the WFR. The participants were encouraged to maintain their normal diet during the recording period, as any change in diet could influence the validation of the FFQ. Every participant received a weighing scale (Swordfish SFKSW14E) and was told to use this rather than a personal weighing scale. The recording booklet contained two pages of information on how to weigh and record food. This information was reviewed, followed by a review of the booklet itself. To accurately record the weight of the food, participants were given boxes to weigh the remains after a meal. A practical example was conducted with weighing and recording of a test meal. When eating out, participants were instructed to weigh and record their food as usual if possible. If the weighing scale was not accessible, participants were instructed to take note of what they ate, take a picture if possible and estimate portion size. The participants were offered a pre-paid envelope if they did not have the opportunity to deliver the recording booklet in person after completing the WFR. The WFR started the following day and continued for seven consecutive days. The participants were encouraged to make contact by e-mail or telephone if they had any questions.

Data entry of the food records was conducted by the corresponding author in collaboration with two research assistants. Calculating food and nutrient intake was overseen by one of the co-authors. Foods and beverages recorded by the participants in the WFR were linked to a corresponding food-code in the Norwegian food composition table [16]. The Norwegian Food Safety Authority’s “Weights, measures and portion sizes for food” was used when converting from volume measurements to grams and for calculating the weight yield factor from cooking [17]. When participants provided recipes, these were replaced with the closest approximate food-code. We used FoodCalc [19] and the Norwegian food composition table [16] to calculate food and nutrient intakes from the WFR. Food and beverage items assessed by the WFR were aggregated into the same 28 non-overlapping food groups used for the FFQ.

### Statistical analysis

Descriptive analyses were used to evaluate the characteristics of the participants (age, height, weight, body mass index (BMI), level of education). Most of the nutrients used to assess reproducibility and relative validity were not normally distributed and presented as median with 25th and 75th percentile, although some were considered to be normally distributed and therefore presented as mean with standard deviation (SD). The food groups used to organize the FFQ test-retest and WFR were not normally distributed, and therefore presented as median with 25th and 75th percentile. We used Spearman’s rank correlation coefficient to examine the correlations for the test-retest reproducibility and the relative validity. Correlation coefficients between 0.5 and 0.7 has shown to be common when testing the reproducibility between two administrations of an FFQ [10], and a Spearman correlation coefficient above 0.5 is recommended for nutrients in dietary validation studies [20].

We also examined energy intake and intake of vegetables and fruits assessed by the FFQ and the WFR by Bland-Altman plots, i.e. by plotting the mean energy intake and intake of vegetables and fruits (x-axis) against their mean difference for each participant [21]. Further, the total intake per day for the food and beverages included in each food group were ranked into tertiles of intake for the FFQ and WFR. The FFQ’s ability to categorize participants into the correct tertile of intake was assessed by calculating the percentage of agreement. Participants were presented as percent correctly classified or grossly misclassified. Participants correctly classified were categorized in the same tertile of intake for both measurements, whereas participants that were grossly misclassified were categorized in non-adjacent tertiles. Unweighted Cohen’s Kappa statistics was analysed for food intake in each food group ranked into tertiles to identify the strength of agreement. Values of Kappa, according to Masson et al., are categorized as follows: < 0.20: poor agreement, 0.21-0.40: fair agreement, 0.41-0.60: moderate agreement, 0.61-0.80: good agreement, and > 0.80: very good agreement [20]. Self-reported height and weight were used to calculate BMI (kg/m^2^). The significance level was set to 5%, and all statistical analysis were carried out using the computer program IBM SPSS Statistics for Windows, version 24 (IBM Corp., Armonk, N.Y., USA).

## Results

### Sample

The characteristics of the 29 participants who completed the first FFQ are presented in Table 1. There was an uneven distribution of women and men in the study. Median age of the participants was 23 years while median BMI was 24.1 (kg/m^2^). The majority of the sample had a higher level of education (university/college up to 4 years or more). The 21 participants (17 women and 4 men) that completed both the test-retest of the FFQ and the WFR were used to assess reproducibility and relative validity of the FFQ.

**Table 1:** Descriptive statistics of the participants that signed up to the reproducibility and validation study (*n* = 29)

	Study population	Women	Men
Number of participants, *n* (%)	29 (100)	21 (72)	8 (28)
Median age, y	23 (22, 27)	24 (22, 28)	23 (22, 24)
Median height, cm	170 (164, 178)	167 (161, 170)	183 (178, 193)
Median weight, kg	68 (64, 80)	66 (62, 70)	87 (68, 100)
Median BMI, kg/m^2^	24.1 (21.7, 25.8)	24.1 (21.5, 25.5)	24.6 (22.0, 26.1)
Education, *n* (%)			
- High School	6 (21)	5 (24)	1 (12)
- Vocational education	3 (10)	3 (14)	0 (0)
- University/college up to 4 years	11 (38)	5 (24)	6 (75)
- University/college more than 4 years	9 (31)	8 (38)	1 (12)

Presented as frequency (percentage) and median (inter quartile range reported as the 25th percentile, 75th percentile). *BMI* Body mass index

### Test-retest reproducibility

The median correlation coefficient for energy and nutrients was 0.85, ranging from *r =* 0.56 for vitamin D to *r =* 0.93 for calcium. There were 15 nutrients that showed high correlation (> 0.7) and two nutrients that were in the common range of correlation for reproducibility (0.5 – 0.7) (Table 2). The median Spearman’s rank correlation coefficient for the food groups was 0.75, ranging from *r* = 0.22 for processed red meat to *r* = 0.93 for oils, butter and margarine (Table 3). There were 19 food groups that showed high correlation, five food groups in the common range of correlation for reproducibility, and four food groups showed a low degree of correlation. For all but the food groups processed red meat, liver-pate, fatty fish, fish dishes, rice, pasta and noodle, and salty snacks, less than 10% of the participants were grossly misclassified, that is, classified into a non-adjacent tertile. The median Cohen’s Kappa value for the food groups was 0.51, with a range from *k* = 0.01 for processed red meat to *k* = 0.78 for low fibre bread and crispbread.

**Table 2:** Test-retest reproducibility and relative validity of energy and nutrients for the food-frequency questionnaire (*n* = 21)

	FFQ 1		Test-retest reproducibility (FFQ 2)			*P*	Relative validity (WFR)			*P*
	Median	(P25, P75)	Median	(P25, P75)	Spearman’s *r*		Median	(P25, P75)	Spearman’s *r*	
Energy (kcal/day)	2310	(1478, 2745)	1921	(1619, 2796)	0.82	**< 0.001**	2170	(1834, 3042)	0.51	**0.018**
Energy (kJ/day)	9696	(6194, 11,519)	8086	(6795, 11,722)	0.82	**< 0.001**	9079	(7672, 12,801)	0.51	**0.018**
Fat (g/day)	82^a^	(24)	71	(59, 109)	0.71	**< 0.001**	93^a^	(26)	0.37^b^	0.104
Saturated fat (g/day)	25	(22, 35)	25	(20, 37)	0.77	**< 0.001**	34	(26, 40)	< 0.05	0.832
Carbohydrates (g/day)	261	(154, 300)	231	(177, 302)	0.86	**< 0.001**	250	(175, 386)	0.59	**0.005**
Fibre (g/day)	30	(17, 38)	29	(19, 38)	0.85	**< 0.001**	33^a^	(13)	0.70	**< 0.001**
Protein (g/day)	92	(66, 127)	96	(69, 119)	0.87	**< 0.001**	98	(71, 110)	0.50	**0.021**
Alcohol (g/day)	2.8	(0.3, 7.5)	3.8	(1.1, 5.1)	0.83	**< 0.001**	0	(0, 6.6)	0.71	**< 0.001**
Vitamin A (RAE/day)	779	(349, 979)	683	(506, 975)	0.87	**< 0.001**	813	(569, 1356)	0.72	**< 0.001**
Folate (μg/day)	312^a^	(135)	298	(199, 416)	0.90	**< 0.001**	317	(254, 436)	0.78	**< 0.001**
Vitamin B12 (μg/day)	6.1	(3.7, 8.4)	6.8^a^	(2.8)	0.86	**< 0.001**	5.2	(4.3, 6.6)	0.66	**0.001**
Calcium (mg/day)	811	(623, 1197)	828	(497, 1373)	0.93	**< 0.001**	1122	(774, 1280)	0.55	**0.010**
Iron (mg/day)	12^a^	(4.4)	11^a^	(4.1)	0.85^b^	**< 0.001**	12	(9.8, 17)	0.67	**0.001**
Iodine (μg/day)	126	(94, 228)	142	(95, 221)	0.88	**< 0.001**	131	(97, 161)	0.67	**0.001**
Vitamin D (μg/day)	5.6	(3.3, 7.4)	5.5	(3.3, 7.7)	0.56	**0.008**	3.9	(2.8, 5.6)	0.46	**0.034**
Salt (g/day)	7.7	(5.2, 8.9)	6.1	(5.2, 10)	0.85	**< 0.001**	6.6	(5.6, 9.0)	0.21	0.363
Sugars (g/day)	20	(16, 27)	27	(15, 38)	0.59	**0.005**	31	(19, 67)	0.62	**0.003**

Energy and nutrient intake for food-frequency questionnaire 1 (FFQ), food-frequency questionnaire 2 and 7-day weighed food record (WFR). Energy and nutrients presented as median (25th percentile, 75th percentile), Spearman’s rank correlation coefficient and p-value. Vitamin A presented as retinol activity equivalents (RAE = sum retinol + 1/12 beta-carotene). Statistically significant *p*-values marked in **bold**

^a^Mean (standard deviation)

^b^Pearson’s correlation coefficient

**Table 3:** Test-retest reproducibility of food groups for the food-frequency questionnaire (*n* = 21)

	FFQ 1		FFQ 2		Spearman’s *r*	*P*	CC %	GM %	Kappa *k*	*P*
	Median	(P25, P75)	Median	(P25, P75)						
Milk, yoghurt, milk dessert (ml/day)	184	(103, 335)	168	(54, 266)	0.81	**< 0.001**	62	0	0.43	**0.006**
Cheese (g/day)	20	(10, 31)	18	(2.9, 35)	0.82	**< 0.001**	67	0	0.50	**0.001**
Eggs (g/day)	18	(16, 54)	18	(12, 66)	0.90	**< 0.001**	67	0	0.51	**< 0.001**
Poultry (g/day)	22	(11, 54)	21	(11, 31)	0.76	**< 0.001**	71	5	0.57	**< 0.001**
Red meat, non-processed (g/day)	12	(10, 20)	20	(8.5, 28)	0.85	**< 0.001**	62	0	0.43	**0.005**
Red meat, processed (g/day)	14	(1.8, 19)	9.3	(1.8, 21)	0.22	0.331	33	14	0.01	0.946
Liver-pate (g/day)	0	(0, 7.1)	2.9	(0, 7.1)	0.69	**0.001**	71	10	0.54	**0.001**
Meat dishes (g/day)	45	(23, 66)	45	(21, 67)	0.66	**0.001**	52	5	0.29	0.063
Fatty fish (g/day)	11	(5.4, 21)	11	(11, 21)	0.40	0.073	71	10	0.56	**< 0.001**
Lean fish (g/day)	14	(0, 29)	14	(0, 14)	0.79	**< 0.001**	71	0	0.56	**< 0.001**
Fish spread (g/day)	7.9	(0, 14)	5.7	(0, 22)	0.70	**< 0.001**	71	5	0.57	**< 0.001**
Fish dishes (g/day)	11	(0, 11)	11	(0, 11)	0.46	**0.035**	43	14	0.23	**0.033**
Breakfast cereal, high grain (g/day)	14	(7.1, 107)	36	(7.1, 100)	0.83	**< 0.001**	71	0	0.57	**< 0.001**
Rice, pasta, noodle (g/day)	76	(46, 113)	76	(43, 133)	0.55	**0.010**	52	10	0.28	0.066
Bread, crispbread, low fibre (g/day)	5.7	(0, 17)	5.7	(0, 23)	0.75	**< 0.001**	86	5	0.78	**< 0.001**
Bread, crispbread, high fibre (g/day)	102	(34, 142)	102	(38, 123)	0.76	**< 0.001**	71	5	0.57	**< 0.001**
Nuts, almonds (g/day)	1.4	(0.7, 7.1)	2.9	(0.7, 7.1)	0.64	**0.002**	71	5	0.56	**< 0.001**
Potatoes (g/day)	33	(20, 68)	31	(15, 72)	0.76	**< 0.001**	62	5	0.43	**0.006**
Vegetables (g/day)	116	(75, 189)	102	(69, 163)	0.84	**< 0.001**	71	5	0.57	**< 0.001**
Fruits (g/day)	172	(50, 249)	133	(58, 304)	0.76	**< 0.001**	67	0	0.50	**0.001**
Sugar, sweets, desserts(g/day)	28	(12, 37)	25	(12, 39)	0.70	**< 0.001**	57	5	0.36	**0.020**
Oils, butter, margarine (g/day)	2.1	(0.4, 16)	2.9	(0, 10)	0.93	**< 0.001**	81	0	0.71	**< 0.001**
Coffee, tea, water (ml/day)	964	(789, 1323)	964	(735, 1196)	0.90	**< 0.001**	76	0	0.64	**< 0.001**
Juice, sweet drinks (ml/day)	171	(111, 257)	200	(86, 489)	0.72	**< 0.001**	52	0	0.29	0.060
Alcohol (ml/day)	53	(1.4, 154)	69	(19, 111)	0.83	**< 0.001**	81	5	0.72	**< 0.001**
Pizza (g/day)	43	(21, 43)	21	(21, 43)	0.38	0.088	33	5	0.16	**0.038**
Salty snacks (g/day)	5.7	(2.9, 8.6)	5.7	(2.9, 8.6)	0.58	**0.006**	48	10	0.21	0.156
Mixed dishes (g/day)	84	(79, 173)	84	(70, 154)	0.75	**< 0.001**	67	5	0.49	**0.001**

Food intake (gram/milliliter per day) for food groups (median, 25th and 75th percentile, Spearman’s rank correlation coefficient, percent correctly classified (CC) and grossly misclassified (GM) into tertiles of intake, Cohen’s Kappa value). Statistically significant *p*-values marked in **bold**

### Relative validity (FFQ vs 7-day WFR)

**Fig. 2:**
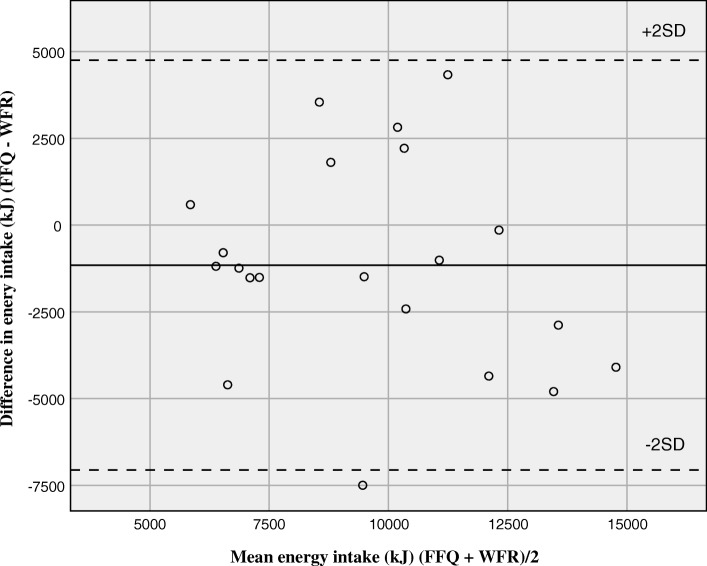
Bland-Altman plot for measuring daily energy intake. Bland-Altman plot between the food frequency questionnaire (FFQ) and the weighed food record (WFR) methods for measuring daily energy intake. The solid line represents the mean difference between the two methods, and the dashed lines represent the limits of agreement corresponding to ±2 (SD)

**Fig. 3:**
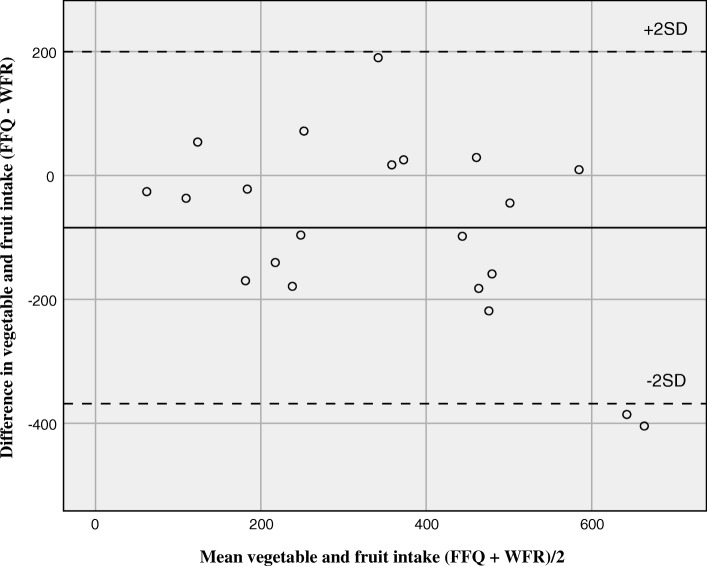
Bland-Altman plot for measuring daily vegetables and fruit intake. Bland-Altman plot between the food frequency questionnaire (FFQ) and the weighed food record (WFR) methods for measuring daily vegetable and fruit intake. The solid line represents the mean difference between the two methods, and the dashed lines represent the limits of agreement corresponding to ±2 (SD)

The calculated daily median (mean) energy intake was 9.7 MJ (mean: 9.1 MJ) by the FFQ and 9.1 MJ (mean: 10.2 MJ) by the WFR. The Bland-Altman plot showed that although the mean difference between the methods (bias) was small, the confidence limits were wide and showed large differences at the individual level (Fig. 2). Energy intake by the FFQ ranged from 4.3 MJ (1028 kcal) to 13.4 MJ (3203 kcal) and by the WFR from 5.5 MJ (1315 kcal) to 9.9 MJ (2366 kcal), all are within a plausible range for young adults. The calculated daily median (mean) intake of vegetable and fruit was 367 g (mean: 311 g) by the FFQ and 350 g (mean: 395 g) by the WFR (Fig. 3). Vegetable and fruit intake by the FFQ ranged from 49 g to 590 g and by the WFR from 75 g to 866 g, all are within a plausible range. The median intakes were higher by the FFQ than by the WFR for energy and four other nutrients (carbohydrates, fibre, sodium, and iron). This was also seen for food groups including vegetables and fruit, processed red meat, fish dishes, high fibre bread and crispbread, sugar, sweets and desserts, and pizza. Still, the calculated mean intakes were lower by the FFQ than by the WFR, as illustrated by the Bland-Altman plots (Figs. 2 and 3).

Correlations for energy and nutrients had a median of 0.59, with a range from *r =* < 0.05 for saturated fat to *r =* 0.78 for folate. There were 13 nutrients that showed moderate to good correlation (> 0.5) and four nutrients that were below the recommended correlation coefficient for relative validation (Table 2). Spearman’s rank correlation coefficient, percentage of agreement and Cohen’s Kappa value were analysed for food groups to investigate the relative validity (Table 4). The median Spearman’s rank correlation coefficient for the 28 corresponding food groups was 0.54, ranging from *r* = − 0.14 for potatoes to *r* = 0.81 for oils, butter and margarine. There were 16 food groups that showed moderate to good correlation and 12 food groups with low to moderate correlation. For all but the food groups fatty fish, potatoes and salty snacks, less than 20% of the participants were grossly misclassified. The median Cohen’s Kappa value for the food groups was 0.28, with a range from *k* = − 0.13 for potatoes to *k* = 0.65 for oils, butter and margarine.

**Table 4:** Relative validity of food groups for the food-frequency questionnaire (*n* = 21)

	FFQ 1		WFR		Spearman’s *r*	*P*	CC%	GM%	Kappa *k*	*P*
	Median	(P25, P75)	Median	(P25, P75)						
Milk, yoghurt, milk desserts (ml/day)	184	(103, 335)	222	(117, 424)	0.63	**0.002**	52	0	0.28	0.066
Cheese (g/day)	20	(10, 31)	35	(13, 54)	0.62	**0.003**	62	5	0.43	**0.005**
Eggs (g/day)	18	(16, 54)	21	(7.8, 43)	0.72	**< 0.001**	52	0	0.28	0.061
Poultry (g/day)	22	(11, 54)	23	(3.6, 67)	0.50	**0.020**	52	14	0.29	0.059
Red meat, non-processed (g/day)	12	(10, 20)	18	(7.4, 32)	0.20	0.392	38	14	0.06	0.689
Red meat, processed (g/day)	14	(1.8, 19)	22	(0, 56)	0.40	0.077	38	5	0.06	0.689
Liver-pate (g/day)	0	(0, 7.1)	0	(0, 6.3)	0.71	**< 0.001**	71	5	0.50	**0.002**
Meat dishes (g/day)	45	(23, 66)	0	(0, 0)	0.04	0.873	33	5	< 0.01	1.000
Fatty fish (g/day)	11	(5.4, 21)	0	(0, 17)	0.03	0.891	38	29	0.13	0.301
Lean fish (g/day)	14	(0, 29)	0	(0, 17)	0.10	0.657	19	10	−0.09	0.393
Fish spread (g/day)	7.9	(0, 14)	0	(0, 17)	0.72	**< 0.001**	67	10	0.49	**< 0.001**
Fish dishes (g/day)	11	(0, 11)	0	(0, 42)	0.12	0.621	33	19	0.02	0.869
Breakfast cereal, high grain (g/day)	14	(7.1, 107)	22	(2.6, 77)	0.70	**< 0.001**	57	0	0.36	**0.021**
Rice, pasta, noodle (g/day)	76	(46, 113)	52	(13, 89)	0.15	0.505	43	19	0.14	0.376
Bread, crispbread, low fibre (g/day)	5.7	(0, 17)	68	(34, 87)	0.59	**0.005**	43	5	0.15	0.299
Bread, crispbread, high fibre (g/day)	102	(34, 142)	61	(18, 112)	0.58	**0.005**	43	5	0.13	0.377
Nuts, almonds (g/day)	1.4	(0.7, 7.1)	1.9	(0, 17)	0.53	**0.013**	62	5	0.43	**0.004**
Potatoes (g/day)	33	(20, 68)	0	(0, 21)	−0.14	0.539	24	38	−0.13	0.371
Vegetables (g/day)	116	(75, 189)	193	(120, 297)	0.70	**< 0.001**	52	10	0.29	0.060
Fruits (g/day)	172	(50, 249)	117	(74, 279)	0.71	**< 0.001**	52	0	0.28	0.070
Sugar, sweets, desserts (g/day)	28	(12, 37)	17	(7.4, 41)	0.39	0.081	43	14	0.15	0.339
Oils, butter, margarine (g/day)	2.1	(0.4, 16)	12	(4.7, 22)	0.81	**< 0.001**	76	0	0.65	**< 0.001**
Coffee, tea, water (ml/day)	964	(789, 1323)	1383	(771, 1858)	0.56	**0.009**	52	5	0.29	0.063
Juice, sweet drinks (ml/day)	171	(111, 257)	212	(78, 482)	0.66	**0.001**	52	0	0.29	0.060
Alcohol (ml/day)	53	(1.4, 154)	0	(0, 120)	0.69	**< 0.001**	62	5	0.42	**0.003**
Pizza (g/day)	43	(21, 43)	18	(0, 65)	0.47	**0.032**	62	19	0.37	**0.019**
Salty snacks (g/day)	5.7	(2.9, 8.6)	9.1	(1.9, 21)	0.01	0.956	43	33	0.14	0.350
Mixed dishes (g/day)	84	(79, 173)	15	(0, 38)	0.07	0.774	38	19	0.07	0.635

Food intake (gram/milliliter per day) for the food-frequency questionnaire (FFQ) and the 7-day weighed food record (WFR) for food groups (median, 25th and 75th percentile, Spearman’s rank correlation coefficient, percent correctly classified (CC) and grossly misclassified (GM) into tertiles of intake, Cohen’s Kappa value). Statistically significant *p*-values marked in **bold**

## Discussion

The FFQ developed for assessing preconception diet among young adults showed satisfactory test-retest reproducibility, indicating that the FFQ is suitable for its target group. The relative validity of the FFQ explored against 7-day WFR indicated a fair agreement between the test and reference method.

### Test-retest reproducibility

A median correlation coefficient of 0.85 for energy and nutrients and 0.75 for food groups is considered satisfactory for this newly developed FFQ. In assessing reproducibility, repeated measures 1 month or less apart has shown a slightly higher correlation compared with those administered 6 months to 1 year apart [10]. The test and retest of the present FFQ were administered over a period of 19 days. When conducting the retest of the FFQ, it is not wise to re-administer it at a very short interval, because subjects may remember their previous answers [10], although remembering their responses to an FFQ containing 146 questions is unlikely. The WFR was conducted in the time period between test and retest of the FFQ. This may have made participants more aware of what they ate during the subsequent retest of the FFQ, and thereby contributed to the accuracy of the FFQs reproducibility. Our results are similar to other studies that have tested the reproducibility of an FFQ. Macedo-Ojeda et al. tested the reproducibility of a semi-quantitative FFQ assessing intake in food groups and nutrients [22]. Of the 12 food groups included in the study, 10 were comparable with our study. Our results showed somewhat higher correlation coefficients for most food groups, although the foods included in the food groups may differ between the studies. Further, Macedo-Ojeda et al. investigated unadjusted correlations for energy and 26 nutrients, which ranged from *r* = 0.18 for vitamin E to *r* = 0.73 for vitamin B12, compared to our correlations for energy and 15 nutrients which ranged from *r* = 0.56 for vitamin D to *r* = 0.93 for calcium. The study included a wider age group, with a mean age of 27.5 (range 18-71). The reproducibility of an online semi-quantitative FFQ to be used for personalized dietary advice was tested by Fallaize et al. [23]. Their unadjusted correlations for energy and nutrients show results that are similar to ours. The reproducibility of 35 food groups showed a mean correlation coefficient of 0.75, similar to our results. The study had similar percentage of exact agreement for food groups, although their intake was ranked into quartiles. The study sample included a somewhat wider age group with a mean age of 32 (SD 12). Hebden et al. tested the reproducibility of a semi-quantitative FFQ on participants 18-34 years old [24]. The study only had food groups for fruit, fruit including fruit juice, and vegetable. The weighted Kappa value for vegetable servings were similar to our unweighted kappa results, but showed a higher value for fruit servings. Their food groups were ranked into quintiles instead of tertiles.

### Relative validity

Comparison of intake between the FFQ and WFR resulted in a median correlation coefficient of 0.59 for energy and nutrients and 0.54 for food groups. The median Cohen’s Kappa value was 0.28 for the food groups. This indicates fair agreement between the two methods. This is also evident from the Bland-Altman plots (Figs. 2 and 3). Participants being correctly classified into the same tertile of intake ranged from 19% for the food groups lean fish to 76% for oil, butter and margarine. Three of the food groups in the relative validation had 20% or more participants grossly misclassified. The WFR dietary assessment does not reflect the same four-week time span as the FFQ, which it ideally should [10]. Although the WFR assessment covered 7 days in our study, the mean difference in calculated energy intake was relatively low (~ 13%). The differences between mean intakes calculated by the FFQ and WFR were negative (Figs. 2 and 3), indicating that intakes were skewed to the left, i.e. wider range of low than high intakes. As most nutrient intakes correlate strongly with energy intake, energy intake is commonly used to identify and exclude individuals with invalid dietary reports. There are no established limits for plausible energy intakes by FFQs. Without further justification than being implausible, a lower limit of 2.5 MJ/day (600 kcal) and an upper limit of 15 MJ/day (3600 kcal) has been applied as cut-offs for reported energy intake by FFQs in several observational studies [25–27]. All the reported energy intakes in the current study were well within these limits and were considered plausible for young adults. The level of underreporting has in some studies been as high as 46% for women and 29% for men [10].

Steinemann et al. [28] examined the relative validity of an FFQ to be used for estimating the food intake in an adult population. The study sample (*n* = 56) had a mean age of 40 years (range 22-85) and completed a 4-day weighted food record. Correlations were reported for energy, four nutrients and 25 food groups. Our results showed similar correlations as in that study for protein and fat, but higher correlations for energy, carbohydrates and fibre. Comparing the relative validity of food groups, our study had a higher median correlation coefficient than Steinemann et al. The present study had higher correlation coefficients for fruit and vegetable intake compared with Hebden et al. [24]. Our Kappa values for fruit and vegetable intake were somewhat lower than their weighted Kappa, despite our intake being ranked into tertiles instead of quintiles. The relative validity of energy, protein, sugars and alcohol showed similar results to our study, although our results had lower correlation for saturated fat and higher correlations for carbohydrates and dietary fibre compared to Hebden et al. Fallaize et al. compared nutrients and food groups from an FFQ with a 4-day WFR on a sample with a mean age of 26.9 (SD 8.4) [23]. The correlations of 30 nutrients (of which seven were E%) ranged from 0.23 for vitamin D to 0.65 for protein, E%, with a mean value of 0.47. Our results show a wider range with a somewhat higher median correlation value. The correlations for 35 food groups showed similar range as in our study, although our median correlation value was higher. Their results ranged from 18 to 55% of participants being correctly classified into quartiles of intake with a mean of 5% grossly misclassified, which is comparable to our 28 food groups ranked into tertiles.

The nutrients included in our analysis were based on the Norwegian Directorate of Health “A healthy lifestyle before and during pregnancy” [29], which highlights folate, vitamin D, iron, calcium, iodine and vitamin B12. Energy, macronutrients and fibre were included to assess dietary contributions, alcohol was included to asses alcohol intake and vitamin A was included because of its importance in fetus development during pregnancy [30]. Salt and sugar were included as they are important factors in a public health perspective, and are priority areas in the Norwegian “Partnership for a healthier diet” [31]. The study was promoted at a university and on social media related to this university, which may have resulted in a majority of student participants. The time of the implementation of the study coincided with the end of the year-examination for students. Four participants (19%) pointed out that this had interfered with their dietary habits. The upcoming Christmas time was mentioned as a reason for increased consumption of unhealthy foods by five participants (24%). The study sample size is a limitation. We did not conduct an a priori sample size calculation. A reasonable sample size for reproducibility and validation studies is 100-200 persons [10]. The present sample’s gender distribution, with an overrepresentation of women, students and high level of education, limits its representativeness for the population. Evidence have shown that women are more likely to underestimate intake, which may have affected the results [8]. There were 16 of the 21 participants used in the analysis of the FFQ that reported using dietary supplements, of which 12 (75%) of these also reported using dietary supplements in the WFR. There is limited available research on diet in the preconception age group. A scoping review identified a paucity of longitudinal data into the mid and late twenties, a varying use and quality of dietary assessment methods, and a large variety of macronutrients and food groups studied [32].

## Conclusion

This food-frequency questionnaire (FFQ) developed for assessing preconception diet in young adults had satisfactory test-retest reproducibility and fair relative validity compared with 7-day weighed food records. The validated FFQ will be used to investigate transgenerational diet-disease associations in future studies.

## Supplementary information

**Additional file 1.** Diet today – health of tomorrow, food-frequency questionnaire. The English version of the food-frequency questionnaire developed and tested in the study.

## Data Availability

The datasets used and analysed during the current study are available from the corresponding author on reasonable request.

## Abbreviations

BMI: Body mass index
CC: Correctly classified
FFQ: Food-frequency questionnaire
GM: Grossly misclassified
NCD: Non-communicable disease
RAE: Retinol activity equivalents
SD: Standard deviation
WFR: Weighed food record
