# Supplementary material for: Reproducibility and relative validity of a newly developed web-based food-frequency questionnaire for assessment of preconception diet
Source: BMC Nutr. 2019 Nov 7;5:47. doi: 10.1186/s40795-019-0310-y (PMC7050769; doi:10.1186/s40795-019-0310-y)
Supplement: Supplementary file 1 — Additional file 1. Diet today – health of tomorrow, food-frequency questionnaire. The English version of the food-frequency questionnaire developed and tested in the study. [file 40795_2019_310_MOESM1_ESM.pdf]

Supplementary file 1
Diet today – health of tomorrow, food-frequency questionnaire

Hi!
Thank you for participating in this survey! Your participation is important to us. Please fill out the questionnaire as accurately as you can.

The questionnaire concerns what you eat and drink.
Please think back on the previous four weeks, and we will ask what you ate and drank in this period, as well as your mealtime habits.
At the end of the questionnaire there are some questions about physical activity, screen time, sleep and other habits.
Completing the questionnaire will take approximately 25 minutes.
Thank you for participating!

WE WOULD LIKE TO KNOW SOME THINGS ABOUT YOU

1. How old are you?
(age)

______

2. Gender
(1) ❑ Female
(2) ❑ Male

3. Height
(in cm)

______

4. Weight
(in kg)

______

5. What level of education do you have?
Choose the highest completed education
(1) ❑ Less than 9/10 years of primary school
(2) ❑ Primary school
(3) ❑ High school
(4) ❑ Vocational education

(5) ❑ University/college up to 4 years
(6) ❑ University/college more than 4 years
(7) ❑ Other education

WE WOULD LIKE TO KNOW WHAT YOU ATE AND DRANK IN THE PAST 4 WEEKS

DRINKS

1. How often did you drink the following?

1 glass = approximately 2 dl

	**Never**	**1-3 glasses per month**	**1-3 glasses per week**	**4-6 glasses per week**	**1 glass per day**	**2-3 glasses per day**	**More than 3 glasses a day**
Whole milk (sweet/sour, e.g. Kefir)	(1) ❑	(2) ❑	(3) ❑	(4) ❑	(5) ❑	(6) ❑	(7) ❑
Low-fat milk	(1) ❑	(2) ❑	(3) ❑	(4) ❑	(5) ❑	(6) ❑	(7) ❑
Extra skimmed milk	(1) ❑	(2) ❑	(3) ❑	(4) ❑	(5) ❑	(6) ❑	(7) ❑
Skimmed milk	(1) ❑	(2) ❑	(3) ❑	(4) ❑	(5) ❑	(6) ❑	(7) ❑
Soy milk, rice milk or other type of milk	(1) ❑	(2) ❑	(3) ❑	(4) ❑	(5) ❑	(6) ❑	(7) ❑
Cultured milk products	(1) ❑	(2) ❑	(3) ❑	(4) ❑	(5) ❑	(6) ❑	(7) ❑
Chocolate milk	(1) ❑	(2) ❑	(3) ❑	(4) ❑	(5) ❑	(6) ❑	(7) ❑

2. How often did you drink the following?

1 glass = approximately 2 dl

	**Never**	**1-3 glasses per month**	**1-3 glasses per week**	**4-6 glasses per week**	**1 glass per day**	**2-3 glasses per day**	**More than 3 glasses a day**
Tap water, bottled water or mineral water	(1) ❑	(2) ❑	(3) ❑	(4) ❑	(5) ❑	(6) ❑	(7) ❑
Squash, sugar sweetened (e.g. lemonade, Ribena)	(1) ❑	(2) ❑	(3) ❑	(4) ❑	(5) ❑	(6) ❑	(7) ❑
Squash, reduced sugar or sugar-free	(1) ❑	(2) ❑	(3) ❑	(4) ❑	(5) ❑	(6) ❑	(7) ❑

1 glass = approximately 2 dl

	Never	1-3 glasses per month	1-3 glasses per week	4-6 glasses per week	1 glass per day	2-3 glasses per day	More than 3 glasses a day
Orange juice	(1) ❑	(2) ❑	(3) ❑	(4) ❑	(5) ❑	(6) ❑	(7) ❑
Apple juice	(1) ❑	(2) ❑	(3) ❑	(4) ❑	(5) ❑	(6) ❑	(7) ❑
Other juice or nectar (e.g. tropical juice, breakfast juice)	(1) ❑	(2) ❑	(3) ❑	(4) ❑	(5) ❑	(6) ❑	(7) ❑

3. How often did you drink the following?

1 bottle/can = 0,5 liter

	Never	1-3 per month	1-3 per week	4-6 per week	1 per day	2-3 per day	More than 3 a day
Soft drinks (e.g. Coca Cola, Fanta, Sprite)	(1) ❑	(2) ❑	(3) ❑	(4) ❑	(5) ❑	(6) ❑	(7) ❑
Soft drinks, reduced sugar or sugar-free (e.g. Pepsi Max, Sprite Zero, Coca Cola light)	(1) ❑	(2) ❑	(3) ❑	(4) ❑	(5) ❑	(6) ❑	(7) ❑
Sports drink (e.g. Powerade, Gatorade)	(1) ❑	(2) ❑	(3) ❑	(4) ❑	(5) ❑	(6) ❑	(7) ❑
Energy drinks (e.g. Red Bull, Battery, Pure Rush, Cult, Burn)	(1) ❑	(2) ❑	(3) ❑	(4) ❑	(5) ❑	(6) ❑	(7) ❑

4. How often did you drink the following?

	Never	1-3 cups per month	1-3 cups per week	4-6 cups per week	1 cup per day	2-3 cups per day	More than 3 cups a day
Coffee, black	(1) ❑	(2) ❑	(3) ❑	(4) ❑	(5) ❑	(6) ❑	(7) ❑
Cafe latte, cappuccino or other coffee with milk	(1) ❑	(2) ❑	(3) ❑	(4) ❑	(5) ❑	(6) ❑	(7) ❑
Frappuccino, mocaccino, ice coffee or the like	(1) ❑	(2) ❑	(3) ❑	(4) ❑	(5) ❑	(6) ❑	(7) ❑

	Never	1-3 cups per month	1-3 cups per week	4-6 cups per week	1 cup per day	2-3 cups per day	More than 3 cups a day
Tea	(1) ❑	(2) ❑	(3) ❑	(4) ❑	(5) ❑	(6) ❑	(7) ❑

5. How many teaspoons of sugar did you add to your coffee and/or tea?

(1) ❑ None
(2) ❑ 1-3 teaspoons per month
(3) ❑ 1 teaspoon per week
(4) ❑ 2-3 teaspoons per week
(5) ❑ 4-6 teaspoons per week
(6) ❑ 1 teaspoon or more per day

6. How many teaspoons of artificial sweetening (e.g. Splenda) did you add to your coffee and/or tea?

(1) ❑ None
(2) ❑ 1-3 teaspoons per month
(3) ❑ 1 teaspoon per week
(4) ❑ 2-3 teaspoons per week
(5) ❑ 4-6 teaspoons per week
(6) ❑ 1 teaspoon or more per day

How often did you drink the following?

7. Alcohol and alcohol-free beverages

				Weekdays			
	**Do not drink**	**1-3 per month**	**1-3 per week**	**4-6 per week**	**1 a day**	**2-3 a day**	**More than 3 a day**
Non-alcoholic beer, root beer, light beer (0,5 liter)	(1) ❑	(2) ❑	(3) ❑	(4) ❑	(5) ❑	(6) ❑	(7) ❑
Beer (0,5 liter)	(1) ❑	(2) ❑	(3) ❑	(4) ❑	(5) ❑	(6) ❑	(7) ❑
Cider (0,5 liter)	(1) ❑	(2) ❑	(3) ❑	(4) ❑	(5) ❑	(6) ❑	(7) ❑
Alcopop	(1) ❑	(2) ❑	(3) ❑	(4) ❑	(5) ❑	(6) ❑	(7) ❑
Wine (1 glass)	(1) ❑	(2) ❑	(3) ❑	(4) ❑	(5) ❑	(6) ❑	(7) ❑
Liquor, liqueur (1 shot)	(1) ❑	(2) ❑	(3) ❑	(4) ❑	(5) ❑	(6) ❑	(7) ❑

8. Alcohol and alcohol-free beverages

	Weekend					
	**Do not drink**	**1-3 per month**	**1-2 per weekend**	**3-4 per weekend**	**5-6 per weekend**	**More than 6 per weekend**
Non-alcoholic beer, root beer, light beer (0,5 liter)	(1) ❑	(2) ❑	(3) ❑	(4) ❑	(5) ❑	(6) ❑
Beer (0,5 liter)	(1) ❑	(2) ❑	(3) ❑	(4) ❑	(5) ❑	(6) ❑
Cider (0,5 liter)	(1) ❑	(2) ❑	(3) ❑	(4) ❑	(5) ❑	(6) ❑
Alcopop	(1) ❑	(2) ❑	(3) ❑	(4) ❑	(5) ❑	(6) ❑
Wine (1 glass)	(1) ❑	(2) ❑	(3) ❑	(4) ❑	(5) ❑	(6) ❑
Liquor, liqueur (1 shot)	(1) ❑	(2) ❑	(3) ❑	(4) ❑	(5) ❑	(6) ❑

YOGHURT
How often did you eat the following?

1. Natural yoghurt

(1) ❑ Never
(2) ❑ 1-3 cups per month
(3) ❑ 1 cup per week
(4) ❑ 2-3 cups per week
(5) ❑ 4-6 cups per week
(6) ❑ 1 cup a day
(7) ❑ More than 1 cup a day

2. Fruit yoghurt/drinking yoghurt, ordinary
E.g. strawberry, melon, pear/banana

(1) ❑ Never
(2) ❑ 1-3 cups per month
(3) ❑ 1 cup per week
(4) ❑ 2-3 cups per week
(5) ❑ 4-6 cups per week
(6) ❑ 1 cup a day
(7) ❑ More than 1 cup a day

3. Fruit yoghurt/drinking yoghurt, sugar free/reduced sugar content

(1) ❑ Never
(2) ❑ 1-3 cups per month
(3) ❑ 1 cup per week
(4) ❑ 2-3 cups per week
(5) ❑ 4-6 cups per week
(6) ❑ 1 cup a day
(7) ❑ More than 1 cup a day

4. Activia/Actimel drinking yoghurt

(1) ❑ Never
(2) ❑ 1-3 cups per month
(3) ❑ 1 cup per week
(4) ❑ 2-3 cups per week
(5) ❑ 4-6 cups per week
(6) ❑ 1 cup a day
(7) ❑ More than 1 cup a day

BREAD AND GRAIN PRODUCTS
How often did you eat the following?

1. Cornflakes, All-Bran, Special K, Cheerios Oat Crunch or the like

(1) ❑ Never
(2) ❑ 1-3 bowls per month
(3) ❑ 1 bowl per week
(4) ❑ 2-3 bowls per week
(5) ❑ 4-6 bowls per week
(6) ❑ 1 bowl a day
(7) ❑ More than 1 bowl a day

2. Oatmeal/oat porridge
E.g. Quaker Oats, Kellogg's

(1) ❑ Never
(2) ❑ 1-3 bowls per month
(3) ❑ 1 bowl per week
(4) ❑ 2-3 bowls per week
(5) ❑ 4-6 bowls per week
(6) ❑ 1 bowl a day
(7) ❑ More than 1 bowl a day

3. Muesli

E.g. Nestlé Muesli, Kellogg Muesli

(1) ❑ Never
(2) ❑ 1-3 bowls per month
(3) ❑ 1 bowl per week
(4) ❑ 2-3 bowls per week
(5) ❑ 4-6 bowls per week
(6) ❑ 1 bowl a day
(7) ❑ More than 1 bowl a day

How often did you eat the following?

4. White bread, bread with low fibre content

E.g. baguette, pita bread, plain white bread, white rolls

(1) ❑ Never
(2) ❑ 1 slice per week
(3) ❑ 2-4 slices per week
(4) ❑ 5-7 slices per week
(5) ❑ 2-3 slices a day
(6) ❑ More than 3 slices a day

5. Whole wheat bread/wheat bread, bread with a medium fibre content

E.g. rolls, baguette, Kneipp

(1) ❑ Never
(2) ❑ 1 slice per week
(3) ❑ 2-4 slices per week
(4) ❑ 5-7 slices per week
(5) ❑ 2-3 slices a day
(6) ❑ More than 3 slices a day

6. Bread with a high fibre content

E.g. rye bread, whole wheat bread/rolls

(1) ❑ Never
(2) ❑ 1 slice per week
(3) ❑ 2-4 slices per week
(4) ❑ 5-7 slices per week
(5) ❑ 2-3 slices per a
(6) ❑ More than 3 slices a day

How often did you eat the following?

7. Crispbread, low fibre content

(1) ❑ Never
(2) ❑ 1-7 pieces per month
(3) ❑ 2-4 pieces per week
(4) ❑ 5-7 pieces per week
(5) ❑ 2-3 pieces a day
(6) ❑ More than 3 pieces a day

8. Crispbread, high fibre content

(1) ❑ Never
(2) ❑ 1-7 pieces per month
(3) ❑ 2-4 pieces per week
(4) ❑ 5-7 pieces per week
(5) ❑ 2-3 pieces a day
(6) ❑ More than 3 pieces a day

9. How often did you use butter or the like on bread/crispbread?
Made from dairy

(1) ❑ Never
(2) ❑ Rarely
(3) ❑ 1-3 pieces per week
(4) ❑ 4-6 pieces per week
(5) ❑ 1-3 pieces a day
(6) ❑ 4-6 pieces a day
(7) ❑ More than 6 pieces a day

10. How often did you use margarine or the like on bread/crispbread?
Typically produced from vegetable oils

(1) ❑ Never
(2) ❑ Rarely
(3) ❑ 1-3 pieces per week
(4) ❑ 4-6 pieces per week
(5) ❑ 1-3 pieces a day
(6) ❑ 4-6 pieces a day
(7) ❑ More than 6 pieces a day

BREAD TOPPINGS AND SPREADS
On how many pieces of bread/crispbread did you eat the following?

1. White/yellow cheese

(1) ❑ Never
(2) ❑ 1 piece per week
(3) ❑ 2-3 pieces per week
(4) ❑ 4-6 pieces per week
(5) ❑ 1 piece a day
(6) ❑ 2-3 pieces a day
(7) ❑ More than 3 pieces a day

2. Brown cheese

(1) ❑ Never
(2) ❑ 1 piece per week
(3) ❑ 2-3 pieces per week
(4) ❑ 4-6 pieces per week
(5) ❑ 1 piece a day
(6) ❑ 2-3 pieces a day
(7) ❑ More than 3 pieces a day

3. Cream cheese spread
E.g. with ham or prawn, Philadelphia cream cheese

(1) ❑ Never
(2) ❑ 1 piece per week
(3) ❑ 2-3 pieces per week
(4) ❑ 4-6 pieces per week
(5) ❑ 1 piece a day
(6) ❑ 2-3 pieces a day
(7) ❑ More than 3 pieces a day

4. Liver pâté
E.g. baked liver pâté, chicken liver pâté, liver pâté with bacon

(1) ❑ Never
(2) ❑ 1 piece per week
(3) ❑ 2-3 pieces per week
(4) ❑ 4-6 pieces per week
(5) ❑ 1 piece a day
(6) ❑ 2-3 pieces a day
(7) ❑ More than 3 pieces a day

On how many pieces of bread/crispbread did you eat the following?

5. Ham, roast beef or the like

(1) ❑ Never
(2) ❑ 1 piece per week
(3) ❑ 2-3 pieces per week
(4) ❑ 4-6 pieces per week
(5) ❑ 1 piece a day
(6) ❑ 2-3 pieces a day
(7) ❑ More than 3 pieces a day

6. Salami, boiled sausage slices, cured meats or the like

(1) ❑ Never
(2) ❑ 1 piece per week
(3) ❑ 2-3 pieces per week
(4) ❑ 4-6 pieces per week
(5) ❑ 1 piece a day
(6) ❑ 2-3 pieces a day
(7) ❑ More than 3 pieces a day

7. Chicken or turkey cold cuts

(1) ❑ Never
(2) ❑ 1 piece per week
(3) ❑ 2-3 pieces per week
(4) ❑ 4-6 pieces per week
(5) ❑ 1 piece a day
(6) ❑ 2-3 pieces a day
(7) ❑ More than 3 pieces a day

On how many pieces of bread/crispbread did you eat the following?

8. Egg
E.g. boiled, fried, scrambled

(1) ❑ Never
(2) ❑ 1 piece per week
(3) ❑ 2-3 pieces per week
(4) ❑ 4-6 pieces per week
(5) ❑ 1 piece a day
(6) ❑ 2-3 pieces a day
(7) ❑ More than 3 pieces a day

9. Sandwich spread

Mayonnaise based spread

(1) ❑ Never
(2) ❑ 1 piece per week
(3) ❑ 2-3 pieces per week
(4) ❑ 4-6 pieces per week
(5) ❑ 1 piece a day
(6) ❑ 2-3 pieces a day
(7) ❑ More than 3 pieces a day

10. Roe

E.g. Caviar, cod's roe, salmon roe

(1) ❑ Never
(2) ❑ 1 piece per week
(3) ❑ 2-3 pieces per week
(4) ❑ 4-6 pieces per week
(5) ❑ 1 piece a day
(6) ❑ 2-3 pieces a day
(7) ❑ More than 3 pieces a day

11. Fish spread or cold cuts

E.g. mackerel filet with tomato sauce, tuna, herring, smoked salmon

(1) ❑ Never
(2) ❑ 1 piece per week
(3) ❑ 2-3 pieces per week
(4) ❑ 4-6 pieces per week
(5) ❑ 1 piece a day
(6) ❑ 2-3 pieces a day
(7) ❑ More than 3 pieces a day

On how many pieces of bread/crispbread did you eat the following?

12. Chocolate or nut spread, regular type

E.g. Milky-Way, Nutella

(1) ❑ Never
(2) ❑ 1 piece per week
(3) ❑ 2-3 pieces per week
(4) ❑ 4-6 pieces per week
(5) ❑ 1 piece a day
(6) ❑ 2-3 pieces a day

(7) ❑ More than 3 pieces a day

13. Chocolate or nut spread, reduced sugar content

(1) ❑ Never
(2) ❑ 1 piece per week
(3) ❑ 2-3 pieces per week
(4) ❑ 4-6 pieces per week
(5) ❑ 1 piece a day
(6) ❑ 2-3 pieces a day
(7) ❑ More than 3 pieces a day

14. Jam, regular

(1) ❑ Never
(2) ❑ 1 piece per week
(3) ❑ 2-3 pieces per week
(4) ❑ 4-6 pieces per week
(5) ❑ 1 piece a day
(6) ❑ 2-3 pieces a day
(7) ❑ More than 3 pieces a day

15. Jam, reduced sugar content

(1) ❑ Never
(2) ❑ 1 piece per week
(3) ❑ 2-3 pieces per week
(4) ❑ 4-6 pieces per week
(5) ❑ 1 piece a day
(6) ❑ 2-3 pieces a day
(7) ❑ More than 3 pieces a day

On how many pieces of bread/crispbread did you eat the following?

16. Honey

(1) ❑ Never
(2) ❑ 1 piece per week
(3) ❑ 2-3 pieces per week
(4) ❑ 4-6 pieces per week
(5) ❑ 1 piece a day
(6) ❑ 2-3 pieces a day
(7) ❑ More than 3 pieces a day

17. Peanut butter

(1) ❑ Never
(2) ❑ 1 piece per week
(3) ❑ 2-3 pieces per week
(4) ❑ 4-6 pieces per week
(5) ❑ 1 piece a day
(6) ❑ 2-3 pieces a day
(7) ❑ More than 3 pieces a day

MAIN COURSE - DINNER

How often did you eat the following?

1. Meatballs/patties

(1) ❑ Never
(2) ❑ 1-3 times per month
(3) ❑ 1 time per week
(4) ❑ 2-4 times per week
(5) ❑ More than 4 times per week

2. Sausages (of pork and/or beef)

E.g. Wiener sausage, hotdog, bratwurst

(1) ❑ Never
(2) ❑ 1-3 times per month
(3) ❑ 1 time per week
(4) ❑ 2-4 times per week
(5) ❑ More than 4 times per week

3. Pork

E.g. roast, fillet, chop

(1) ❑ Never
(2) ❑ 1-3 times per month
(3) ❑ 1 time per week
(4) ❑ 2-4 times per week
(5) ❑ More than 4 times per week

4. Beef, lamb

E.g. steak, roast, leg, chop

(1) ❑ Never
(2) ❑ 1-3 times per month
(3) ❑ 1 time per week

(4) ❑ 2-4 times per week
(5) ❑ More than 4 times per week

5. Taco (tacos or mince wraps)
(1) ❑ Never
(2) ❑ 1-3 times per month
(3) ❑ 1 time per week
(4) ❑ 2-4 times per week
(5) ❑ More than 4 times per week

How often did you eat the following?

6. Hamburger
(1) ❑ Never
(2) ❑ 1-3 times per month
(3) ❑ 1 time per week
(4) ❑ 2-4 times per week
(5) ❑ More than 4 times per week

7. Pizza
(1) ❑ Never
(2) ❑ 1-3 times per month
(3) ❑ 1 time per week
(4) ❑ 2-4 times per week
(5) ❑ More than 4 times per week

8. Casserole dish
E.g. risotto, stew, casserole with meat
(1) ❑ Never
(2) ❑ 1-3 times per month
(3) ❑ 1 time per week
(4) ❑ 2-4 times per week
(5) ❑ More than 4 times per week

9. Pasta dish with meat
E.g. lasagna, spaghetti with meat sauce
(1) ❑ Never
(2) ❑ 1-3 times per month
(3) ❑ 1 time per week

(4) ❑ 2-4 times per week
(5) ❑ More than 4 times per week

How often did you eat the following?

10. Chicken or turkey
E.g. grilled, fillet, leg
(1) ❑ Never
(2) ❑ 1-3 times per month
(3) ❑ 1 time per week
(4) ❑ 2-4 times per week
(5) ❑ More than 4 times per week

11. Processed chicken products
E.g. Nuggets, clubs, wings, burger, sausages
(1) ❑ Never
(2) ❑ 1-3 times per month
(3) ❑ 1 time per week
(4) ❑ 2-4 times per week
(5) ❑ More than 4 times per week

12. Egg
E.g. fried or omelette
(1) ❑ Never
(2) ❑ 1-3 times per month
(3) ❑ 1 time per week
(4) ❑ 2-4 times per week
(5) ❑ More than 4 times per week

13. Pie with meat or vegetables
(1) ❑ Never
(2) ❑ 1-3 times per month
(3) ❑ 1 time per week
(4) ❑ 2-4 times per week
(5) ❑ More than 4 times per week

How often did you eat the following?

14. Oily fish

E.g. salmon, trout, mackerel (boiled or fried)

(1) ❑ Never
(2) ❑ 1-3 times per month
(3) ❑ 1 time per week
(4) ❑ 2-4 times per week
(5) ❑ More than 4 times per week

15. White fish

E.g. cod, pollock (boiled or fried)

(1) ❑ Never
(2) ❑ 1-3 times per week
(3) ❑ 1 time per week
(4) ❑ 2-4 times per week
(5) ❑ More than 4 times per week

16. Shellfish

E.g. shrimp, scampi, lobster, crab

(1) ❑ Never
(2) ❑ 1-3 time per month
(3) ❑ 1 time per week
(4) ❑ 2-4 times per week
(5) ❑ More than 4 times per week

17. Processed fish meat

E.g. fish cakes, fish sticks

(1) ❑ Never
(2) ❑ 1-3 times per month
(3) ❑ 1 time per week
(4) ❑ 2-4 times per week
(5) ❑ More than 4 times per week

How often did you eat the following?

18. Dishes with beans, lentils or peas

E.g. falafel, hummus, bean casserole, lentil soup

(1) ❑ Never
(2) ❑ 1-3 times per month
(3) ❑ 1 time per week
(4) ❑ 2-4 times per week

(5) ❑ More than 4 times per week

19. Soup
E.g. tomato soup, vegetable soup
(1) ❑ Never
(2) ❑ 1-3 times per month
(3) ❑ 1 time per week
(4) ❑ 2-4 times per week
(5) ❑ More than 4 times per week

How often did you eat the following?

20. Pancakes
(1) ❑ Never
(2) ❑ 1-3 times per month
(3) ❑ 1 time per week
(4) ❑ 2-4 times per week
(5) ❑ More than 4 times per week

21. Rice porridge
(1) ❑ Never
(2) ❑ 1-3 times per month
(3) ❑ 1 time per week
(4) ❑ 2-4 times per week
(5) ❑ More than 4 times per week

Good job, you are now halfway...
Keep up the good work!

SIDE DISHES
How often did you eat the following?

1. Potatoes
Cooked or mashed
(1) ❑ Never
(2) ❑ 1-3 times per month
(3) ❑ 1 time per week
(4) ❑ 2-4 times per week
(5) ❑ More than 4 times per week

2. French fries

(1) ❑ Never
(2) ❑ 1-3 times per month
(3) ❑ 1 time per week
(4) ❑ 2-4 times per week
(5) ❑ More than 4 times per week

3. Potato salad or gratin potatoes

(1) ❑ Never
(2) ❑ 1-3 times per month
(3) ❑ 1 time per week
(4) ❑ 2-4 times per week
(5) ❑ More than 4 times per week

4. Fried or baked potatoes

(1) ❑ Never
(2) ❑ 1-3 times per month
(3) ❑ 1 time per week
(4) ❑ 2-4 times per week
(5) ❑ More than 4 times per week

How often did you eat the following?

5. Rice, pasta/spaghetti and noodles

	Refined				
	**Never**	**1-3 times per month**	**1 time per week**	**2-4 times per week**	**More than 4 times per week**
Rice	(1) ❑	(2) ❑	(3) ❑	(4) ❑	(5) ❑
Pasta/spaghetti	(1) ❑	(2) ❑	(3) ❑	(4) ❑	(5) ❑
Noodles	(1) ❑	(2) ❑	(3) ❑	(4) ❑	(5) ❑

6. Rice, pasta/spaghetti and noodles

	Whole grain				
	**Never**	**1-3 times per month**	**1 time per week**	**2-4 times per week**	**More than 4 times per week**
Rice	(1) ❑	(2) ❑	(3) ❑	(4) ❑	(5) ❑
Pasta/spaghetti	(1) ❑	(2) ❑	(3) ❑	(4) ❑	(5) ❑
Noodles	(1) ❑	(2) ❑	(3) ❑	(4) ❑	(5) ❑

7. Sauce
E.g. brown sauce, white sauce, bearnaise sauce

(1) ❑ Never
(2) ❑ 1-3 times per month
(3) ❑ 1 time per week
(4) ❑ 2-3 times per week
(5) ❑ 4-6 times per week
(6) ❑ 1 or more times a day

8. Sour cream or cream fraiche, regular type
E.g. dip

(1) ❑ Never
(2) ❑ 1-3 times per month
(3) ❑ 1 time per week
(4) ❑ 2-3 times per week
(5) ❑ 4-6 times per week
(6) ❑ 1 or more times a day

9. Sour cream or cream fraiche, low-fat alternative
E.g. dip

(1) ❑ Never
(2) ❑ 1-3 times per month
(3) ❑ 1 time per week
(4) ❑ 2-3 times per week
(5) ❑ 4-6 times per week
(6) ❑ 1 or more times a day

10. Cottage Cheese
E.g. on bread, as a condiment or snack
(1) ❑ Never
(2) ❑ 1-3 times per month
(3) ❑ 1 time per week
(4) ❑ 2-3 times per week
(5) ❑ 4-6 times per week
(6) ❑ 1 or more times a day

How often did you eat the following?

11. Pesto
(1) ❑ Never
(2) ❑ 1-3 times per month
(3) ❑ 1 time per week
(4) ❑ 2-3 times per week
(5) ❑ 4-6 times per week
(6) ❑ 1 or more times a day

12. Dressing
(1) ❑ Never
(2) ❑ 1-3 times per month
(3) ❑ 1 time per week
(4) ❑ 2-3 times per week
(5) ❑ 4-6 times per week
(6) ❑ 1 or more times a day

13. Ketchup
(1) ❑ Never
(2) ❑ 1-3 times per month
(3) ❑ 1 time per week
(4) ❑ 2-3 times per week
(5) ❑ 4-6 times per week
(6) ❑ 1 or more times a day

14. Mustard
(1) ❑ Never
(2) ❑ 1-3 times per month
(3) ❑ 1 time per week
(4) ❑ 2-3 times per week

(5) ❑ 4-6 times per week
(6) ❑ 1 or more times a day

15. Mayonnaise or remoulade
(1) ❑ Never
(2) ❑ 1-3 times per month
(3) ❑ 1 time per week
(4) ❑ 2-3 times per week
(5) ❑ 4-6 times per week
(6) ❑ 1 or more times a day

16. How many teaspoons of sugar did you add to your dinner meal?
E.g. on rice pudding, pancakes or the like
(1) ❑ None
(2) ❑ 1-3 tablespoons per month
(3) ❑ 1 tablespoon per week
(4) ❑ 2-3 tablespoons per week
(5) ❑ 4-6 tablespoons per week
(6) ❑ 1 tablespoon or more a day

17. How often did you salt your dinner meal while eating?
(1) ❑ Never
(2) ❑ 1-3 times per month
(3) ❑ 1 time per week
(4) ❑ 2-3 times per week
(5) ❑ 4-6 times per week
(6) ❑ 1 or more times a day

FRUIT AND VEGETABLES
How often did you eat the following?

1. Apples (1 apple)
(1) ❑ Never
(2) ❑ 1-3 times per month
(3) ❑ 1 time per week
(4) ❑ 2-3 times per week
(5) ❑ 4-6 times per week
(6) ❑ 1 or more times a day

2. Pear (1 pear)

(1) ❑ Never
(2) ❑ 1-3 times per month
(3) ❑ 1 time per week
(4) ❑ 2-3 times per week
(5) ❑ 4-6 times per week
(6) ❑ 1 or more times a day

3. Banana (1 banana)

(1) ❑ Never
(2) ❑ 1-3 times per month
(3) ❑ 1 time per week
(4) ❑ 2-3 times per week
(5) ❑ 4-6 times per week
(6) ❑ 1 or more times a day

4. Orange, mandarin, clementine, grapefruit (1/2 - 1 orange/mandarin/clementine/grapefruit)

(1) ❑ Never
(2) ❑ 1-3 times per month
(3) ❑ 1 time per week
(4) ❑ 2-3 times per week
(5) ❑ 4-6 times per week
(6) ❑ 1 or more times a day

How often did you eat the following?

5. Nectarine, peach or plum (1 nectarine/peach/plum)

(1) ❑ Never
(2) ❑ 1-3 times per month
(3) ❑ 1 time per week
(4) ❑ 2-3 times per week
(5) ❑ 4-6 times per week
(6) ❑ 1 or more times a day

6. Melon (1 slice)

(1) ❑ Never
(2) ❑ 1-3 times per month
(3) ❑ 1 time per week
(4) ❑ 2-3 times per week
(5) ❑ 4-6 times per week

(6) ❑ 1 or more times a day

7. Kiwi (1 kiwi)

(1) ❑ Never
(2) ❑ 1-3 times per month
(3) ❑ 1 time per week
(4) ❑ 2-3 times per week
(5) ❑ 4-6 times per week
(6) ❑ 1 or more times a day

8. Pineapple, fresh (1 slice)

(1) ❑ Never
(2) ❑ 1-3 times per month
(3) ❑ 1 time per week
(4) ❑ 2-3 times per week
(5) ❑ 4-6 times per week
(6) ❑ 1 or more times a day

How often did you eat the following?

9. Berries, fresh or frozen (1 handful)

(1) ❑ Never
(2) ❑ 1-3 times per month
(3) ❑ 1 time per week
(4) ❑ 2-3 times per week
(5) ❑ 4-6 times per week
(6) ❑ 1 or more times a day

10. Grapes (1 handful)

(1) ❑ Never
(2) ❑ 1-3 times per month
(3) ❑ 1 time per week
(4) ❑ 2-3 times per week
(5) ❑ 4-6 times per week
(6) ❑ 1 or more times a day

11. Raisins (1/2 handful)

(1) ❑ Never
(2) ❑ 1-3 times per month

(3) ❑ 1 time per week
(4) ❑ 2-3 times per week
(5) ❑ 4-6 times per week
(6) ❑ 1 or more times a day

12. Dried fruit (1/2 handful)
E.g. apricot, prunes, dates
(1) ❑ Never
(2) ❑ 1-3 times per month
(3) ❑ 1 time per week
(4) ❑ 2-3 times per week
(5) ❑ 4-6 times per week
(6) ❑ 1 or more times a day

How often did you eat the following?

13. Broccoli (2 flower buds)
(1) ❑ Never
(2) ❑ 1-3 times per month
(3) ❑ 1 time per week
(4) ❑ 2-3 times per week
(5) ❑ 4-6 times per week
(6) ❑ 1 or more times a day

14. Cauliflower (2 flower buds)
(1) ❑ Never
(2) ❑ 1-3 times per month
(3) ❑ 1 time per week
(4) ❑ 2-3 times per week
(5) ❑ 4-6 times per week
(6) ❑ 1 or more times a day

15. Onion, garlic or leek (1 tablespoon)
(1) ❑ Never
(2) ❑ 1-3 times per month
(3) ❑ 1 time per week
(4) ❑ 2-3 times per week
(5) ❑ 4-6 times per week
(6) ❑ 1 or more times a day

16. Avocado (1/2 avocado)

(1) ❑ Never
(2) ❑ 1-3 times per month
(3) ❑ 1 time per week
(4) ❑ 2-3 times per week
(5) ❑ 4-6 times per week
(6) ❑ 1 or more times a day

How often did you eat the following?

17. Maize (1/2 cob = 2 tablespoons)

(1) ❑ Never
(2) ❑ 1-3 times per month
(3) ❑ 1 time per week
(4) ❑ 2-3 times per week
(5) ❑ 4-6 times per week
(6) ❑ 1 or more times a day

18. Mushrooms (1 tablespoon)

(1) ❑ Never
(2) ❑ 1-3 times per month
(3) ❑ 1 time per week
(4) ❑ 2-3 times per week
(5) ❑ 4-6 times per week
(6) ❑ 1 or more times a day

19. Peas (1 tablespoon)

(1) ❑ Never
(2) ❑ 1-3 times per month
(3) ❑ 1 time per week
(4) ❑ 2-3 times per week
(5) ❑ 4-6 times per week
(6) ❑ 1 or more times a day

20. Mixed salad (1 portion)
E.g. iceberg/romaine/lettuce with tomato and cucumber

(1) ❑ Never
(2) ❑ 1-3 times per month
(3) ❑ 1 time per week
(4) ❑ 2-3 times per week

(5) ❑ 4-6 times per week
(6) ❑ 1 or more times a day

How often did you eat the following?

21. Spinach (2 tablespoons)
(1) ❑ Never
(2) ❑ 1-3 times per month
(3) ❑ 1 time per week
(4) ❑ 2-3 times per week
(5) ❑ 4-6 times per week
(6) ❑ 1 or more times a day

22. Green, yellow, orange or red pepper (1 ring)
(1) ❑ Never
(2) ❑ 1-3 times per month
(3) ❑ 1 time per week
(4) ❑ 2-3 times per week
(5) ❑ 4-6 times per week
(6) ❑ 1 or more times a day

23. Carrots (1 carrot)
(1) ❑ Never
(2) ❑ 1-3 times per month
(3) ❑ 1 time per week
(4) ❑ 2-3 times per week
(5) ❑ 4-6 times per week
(6) ❑ 1 or more times a day

How often did you eat the following?

24. Cucumber (about 4-5 cm)
(1) ❑ Never
(2) ❑ 1-3 times per month
(3) ❑ 1 time per week
(4) ❑ 2-3 times per week
(5) ❑ 4-6 times per week
(6) ❑ 1 or more times a day

25. Tomato (1 tomato)

(1) ❑ Never
(2) ❑ 1-3 times per month
(3) ❑ 1 time per week
(4) ❑ 2-3 times per week
(5) ❑ 4-6 times per week
(6) ❑ 1 or more times a day

26. Other vegetables
If yes, which one(s)?

(1) ❑ Yes ______
(2) ❑ No

DESSERT AND CAKES
How often did you eat the following?

1. Ice cream (1 scoop or ice pop)
E.g. vanilla, chocolate, brittle, strawberries

(1) ❑ Never
(2) ❑ 1-3 times per month
(3) ❑ 1 time per week
(4) ❑ 2-3 times per week
(5) ❑ 4-6 times per week
(6) ❑ 1 or more times a day

2. Ice pop (1 ice pop)
Water-based frozen confection, such as sugar water, fruit juice or purée

(1) ❑ Never
(2) ❑ 1-3 times per month
(3) ❑ 1 time per week
(4) ❑ 2-3 times per week
(5) ❑ 4-6 times per week
(7) ❑ 1 or more times a day

3. Pudding, mousse, jelly (1 portion)
E.g. chocolate pudding, almond pudding, caramel pudding, lemon mousse

(1) ❑ Never
(2) ❑ 1-3 times per month
(3) ❑ 1 time per week
(4) ❑ 2-3 times per week

(5) ❑ 4-6 times per week
(6) ❑ 1 or more times a day

4. Rice pudding and rice cream dessert (1 portion)

(1) ❑ Never
(2) ❑ 1-3 times per month
(3) ❑ 1 time per week
(4) ❑ 2-3 times per week
(5) ❑ 4-6 times per week
(6) ❑ 1 or more times a day

How often did you eat the following?

5. Canned fruit (1 portion)
E.g. canned apricots, pears, pineapple, cocktail mix

(1) ❑ Never
(2) ❑ 1-3 times per month
(3) ❑ 1 time per week
(4) ❑ 2-3 times per week
(5) ❑ 4-6 times per week
(6) ❑ 1 or more times a day

6. Pie (1 slice)
E.g. apple pie, blueberry pie, chocolate pie

(1) ❑ Never
(2) ❑ 1-3 times per month
(3) ❑ 1 time per week
(4) ❑ 2-3 times per week
(5) ❑ 4-6 times per week
(6) ❑ 1 or more times a day

7. Cream (1/2 cup)
E.g. as topping for strawberries, cake, hot chocolate

(1) ❑ Never
(2) ❑ 1-3 times per month
(3) ❑ 1 time per week
(4) ❑ 2-3 times per week
(5) ❑ 4-6 times per week
(6) ❑ 1 or more times a day

How often did you eat the following?

8. Custard (1/2 cup)
E.g. as topping for chocolate pudding or hot berries
(1) ❑ Never
(2) ❑ 1-3 times per month
(3) ❑ 1 time per week
(4) ❑ 2-3 times per week
(5) ❑ 4-6 times per week
(6) ❑ 1 or more times a day

9. Pastries (1 piece)
E.g. bun, Danish pastry, sweet roll
(1) ❑ Never
(2) ❑ 1-3 times per month
(3) ❑ 1 time per week
(4) ❑ 2-3 times per week
(5) ❑ 4-6 times per week
(6) ❑ 1 or more times a day

10. Cake (1 piece)
E.g. cream cake, brownie, chocolate cake
(1) ❑ Never
(2) ❑ 1-3 times per month
(3) ❑ 1 time per week
(4) ❑ 2-3 times per week
(5) ❑ 4-6 times per week
(6) ❑ 1 or more times a day

11. Cookies (1 cookie)
E.g. chocolate biscuit, Oreo
(1) ❑ Never
(2) ❑ 1-3 times per month
(3) ❑ 1 time per week
(4) ❑ 2-3 times per week
(5) ❑ 4-6 times per week
(6) ❑ 1 or more times a day

SNACKS

How often did you eat the following?

1. Potato chips, tortilla chips (1 small bag)

(1) ❑ Never
(2) ❑ 1-3 times per month
(3) ❑ 1 time per week
(4) ❑ 2-3 times per week
(5) ❑ 4-6 times per week
(6) ❑ 1 or more times a day

2. Popcorn (1/2 bag)

(1) ❑ Never
(2) ❑ 1-3 times per month
(3) ❑ 1 time per week
(4) ❑ 2-3 times per week
(5) ❑ 4-6 times per week
(6) ❑ 1 or more times a day

3. Nuts (1 handful)

(1) ❑ Never
(2) ❑ 1-3 times per month
(3) ❑ 1 time per week
(4) ❑ 2-3 times per week
(5) ❑ 4-6 times per week
(6) ❑ 1 or more times a day

How often did you eat the following?

4. Candy (1 handful)

E.g. gummy bears, sweets, liquorice, caramels

(1) ❑ Never
(2) ❑ 1-3 times per month
(3) ❑ 1 time per week
(4) ❑ 2-3 times per week
(5) ❑ 4-6 times per week
(6) ❑ 1 or more times a day

5. Vanilla and/or milk chocolate, 6 pieces

(1) ❑ Never
(2) ❑ 1-3 times per month
(3) ❑ 1 time per week
(4) ❑ 2-3 times per week
(5) ❑ 4-6 times per week
(6) ❑ 1 or more times a day

6. Dark chocolate, 6 pieces

(1) ❑ Never
(2) ❑ 1-3 times per month
(3) ❑ 1 time per week
(4) ❑ 2-3 times per week
(5) ❑ 4-6 times per week
(6) ❑ 1 or more times a day

7. Chocolate bar (1 bar)
E.g. Double Decker, Mars, Snickers, Lion

(1) ❑ Never
(2) ❑ 1-3 times per month
(3) ❑ 1 time per week
(4) ❑ 2-3 times per week
(5) ❑ 4-6 times per week
(6) ❑ 1 or more times a day

MEALTIME HABITS

1. How many times during the week did you eat breakfast, lunch, dinner and supper?

	Weekdays			
	**Never or almost never**	**1-2 times per week**	**3-4 times per week**	**Every weekday**
Breakfast	(1) ❑	(2) ❑	(3) ❑	(4) ❑
Lunch	(1) ❑	(2) ❑	(3) ❑	(4) ❑
Dinner	(1) ❑	(2) ❑	(3) ❑	(4) ❑
Supper	(1) ❑	(2) ❑	(3) ❑	(4) ❑

2. How many times during the weekend did you eat breakfast, lunch, dinner and supper?

	Weekend		
	**Never or almost never**	**1 time per weekend**	**Every day during the weekend**
Breakfast	(1) ❑	(2) ❑	(3) ❑
Lunch	(1) ❑	(2) ❑	(3) ❑
Dinner	(1) ❑	(2) ❑	(3) ❑
Supper	(1) ❑	(2) ❑	(3) ❑

3. How often did you eat breakfast or dinner with others (e.g. cohabitant)?

(1) ❑ Never or almost never
(2) ❑ 1-2 times per week
(3) ❑ 3-4 times per week
(4) ❑ 5-6 times per week
(5) ❑ Every day

4. Where did you usually get your lunch from?
Check the category(s) that apply to you

(1) ❑ Eat lunch at home
(2) ❑ Bring packed lunch from home
(3) ❑ Buy at university/college/job
(4) ❑ Buy outside of university/college/job
(5) ❑ Do not eat lunch

5. How often did you eat at a restaurant or "take away"?

(1) ❑ Never
(2) ❑ 1-3 times per month
(3) ❑ 1 time per week
(4) ❑ 2-3 times per week
(5) ❑ 4-6 times per week
(6) ❑ 1 or more times a day

6. How often did you eat from a fast-food restaurant?
E.g. McDonalds, Burger King, petrol station

(1) ❑ Never
(2) ❑ 1-3 times per month
(3) ❑ 1 time per week
(4) ❑ 2-3 times per week

(5) ❑ 4-6 times per week
(6) ❑ 1 or more times a day

7. How often did you take the following?

	Never	1-2 times per month	3-5 times per month	1-3 times per week	4-6 times per week	Every day
Liquid omega-3 (1 tablespoon)	(1) ❑	(2) ❑	(3) ❑	(4) ❑	(5) ❑	(6) ❑
Omega-3 capsules	(1) ❑	(2) ❑	(3) ❑	(4) ❑	(5) ❑	(6) ❑
Multivitamin	(1) ❑	(2) ❑	(3) ❑	(4) ❑	(5) ❑	(6) ❑
Multivitamin with minerals	(1) ❑	(2) ❑	(3) ❑	(4) ❑	(5) ❑	(6) ❑
Iron tablets	(1) ❑	(2) ❑	(3) ❑	(4) ❑	(5) ❑	(6) ❑
Vitamin A	(1) ❑	(2) ❑	(3) ❑	(4) ❑	(5) ❑	(6) ❑
Vitamin C	(1) ❑	(2) ❑	(3) ❑	(4) ❑	(5) ❑	(6) ❑
Vitamin D	(1) ❑	(2) ❑	(3) ❑	(4) ❑	(5) ❑	(6) ❑
Folate	(1) ❑	(2) ❑	(3) ❑	(4) ❑	(5) ❑	(6) ❑
Protein supplement (powder/shake/bar etc.)	(1) ❑	(2) ❑	(3) ❑	(4) ❑	(5) ❑	(6) ❑
Meal replacements (powder/shake/bar etc.)	(1) ❑	(2) ❑	(3) ❑	(4) ❑	(5) ❑	(6) ❑

8. Other
Did you take any supplements other than those above? If yes; what and how often?
(1) ❑ Yes _____
(2) ❑ No

9. Do you have a food allergy?
(1) ❑ Yes
(2) ❑ No

10. Which?
(1) ❑ Milk
(2) ❑ Egg
(3) ❑ Nuts
(4) ❑ Shellfish
(5) ❑ Other _____

11. Is there something you avoid eating?
If yes; what and why?

(1) ❑ Yes _____
(2) ❑ No

12. Have you been on a diet over the past 4 weeks?
If yes; which?

(1) ❑ Yes _____
(2) ❑ No

13. Is the last month typical of what you usually eat?
If no, why not?

(1) ❑ Yes
(2) ❑ No _____

WE WOULD LIKE TO KNOW HOW ACTIVE YOU WERE THE LAST 4 WEEKS

1. How often were you physically active for at least 30 minutes in total during the day?
Physically active meaning all activity where your heart beats faster and you breathe harder and faster than usual, e.g. brisk walking

(1) ❑ Never
(2) ❑ Less than 1 time per week
(3) ❑ 1 time per week
(4) ❑ 2 times per week
(5) ❑ 3 times per week
(6) ❑ 4 times per week
(7) ❑ 5 times per week
(8) ❑ 6 times per week
(9) ❑ Every day

2. How many hours of physical exercise did you do per week?
Systematic physical exercise to develop, improve or maintain skills, abilities and/or attributes

(1) ❑ Never
(2) ❑ 1-2 hours per week
(3) ❑ 3-4 hours per week
(4) ❑ 5-6 hours per week
(5) ❑ 7-8 hours per week

(6) ❑ 8 hours or more per week

WE WOULD LIKE TO KNOW ABOUT YOUR SCREEN TIME OVER THE LAST 4 WEEKS

3. How many hours a day did you tend to watch TV/movies/series/videogames in your spare time (on TV, PC, tablet, mobile etc.)?
Check one box for weekdays and one box for weekend

	None	1 hour	2 hours	3 hours	4 hours	5 hours	6 hours	7 hours	8 hours or more
Weekdays	(1) ❑	(2) ❑	(3) ❑	(4) ❑	(5) ❑	(6) ❑	(7) ❑	(8) ❑	(9) ❑
Weekend	(1) ❑	(2) ❑	(3) ❑	(4) ❑	(5) ❑	(6) ❑	(7) ❑	(8) ❑	(9) ❑

4. How many hours a day did you tend to use PC/tablet/mobile etc. for chatting, surfing the internet, email and the like in your spare time?
Check one box for weekdays and one box for weekend

	None	1 hour	2 hours	3 hours	4 hours	5 hours	6 hours	7 hours	8 hours or more
Weekdays	(1) ❑	(2) ❑	(3) ❑	(4) ❑	(5) ❑	(6) ❑	(7) ❑	(8) ❑	(9) ❑
Weekend	(1) ❑	(2) ❑	(3) ❑	(4) ❑	(5) ❑	(6) ❑	(7) ❑	(8) ❑	(9) ❑

WE WOULD LIKE TO KNOW ABOUT YOUR SLEEP HABITS OVER THE LAST 4 WEEKS

5. How many hours did you sleep each night on weekdays?
(1) ❑ Less than 5 hours
(2) ❑ 5 hours
(3) ❑ 6 hours
(4) ❑ 7 hours
(5) ❑ 8 hours
(6) ❑ 9 hours
(7) ❑ 10 hours or more

6. How many hours did you sleep each night in the weekend?

(1) ❑ Less than 5 hours
(2) ❑ 5 hours
(3) ❑ 6 hours
(4) ❑ 7 hours
(5) ❑ 8 hours
(6) ❑ 9 hours
(7) ❑ 10 hours or more

7. Use of tobacco products

	**Never**	**Rarely**	**Occasionally**	**Daily**
Cigarettes	(1) ❑	(2) ❑	(3) ❑	(4) ❑
Cigarillo	(1) ❑	(2) ❑	(3) ❑	(4) ❑
Cigar	(1) ❑	(2) ❑	(3) ❑	(4) ❑
Pipe	(1) ❑	(2) ❑	(3) ❑	(4) ❑
Electronic cigarette	(1) ❑	(2) ❑	(3) ❑	(4) ❑
Snuff	(1) ❑	(2) ❑	(3) ❑	(4) ❑

THANK YOU FOR PARTICIPATING! :-)

Submit by pressing "Exit".
